# Intervention in Acute Pulmonary Embolism: A Comparison of the 2026 AHA/ACC and 2019 ESC Guidelines

**DOI:** 10.1016/j.jacadv.2026.102957

**Published:** 2026-07-22

**Authors:** Marco Zuin, Gregory Piazza

**Affiliations:** a Department of Translational Medicine, University of Ferrara, Ferrara, Italy; b Department of Cardio-Thoraco-Vascular Sciences and Public Health, University of Padova, Padua, Italy; c PhD Program in Translation Specialistic Medicine "G.B. Morgagni", Curriculum Cardiovascular Sciences, University of Padua, Padua, Italy; d Department of Cardiology, Madre Teresa di Calcutta Hospital, AULSS 6, South Padova Hospitals, Schiavonia, Italy; e Division of Cardiovascular Medicine, Brigham and Women's Hospital, Harvard Medical School, Boston, Massachusetts, USA; f Thrombosis Research Group, Brigham and Women's Hospital, Harvard Medical School, Boston, Massachusetts, USA

**Keywords:** catheter-directed treatment, guidelines, pulmonary embolism, reperfusion

Acute pulmonary embolism (PE) represents a heterogeneous spectrum of presentations ranging from incidental computed tomography findings to sudden cardiac death. In between, exists a broad population of presentations and potential outcomes in which early risk stratification is essential to guide therapy and identify candidates for reperfusion.^1^^,^^2^ The rapid expansion of catheter-based interventions has outpaced the evidence base, especially as applied to this large heterogeneous subset of patients with PE. The 2026 American Heart Association (AHA)/American College of Cardiology (ACC) guidelines introduce a more granular category-based framework,^2^ whereas the European Society of Cardiology (ESC)/European Respiratory Society (ERS) 2019 guidelines has relied on a more traditional hemodynamic and risk score-based stratification (Figure 1).^1^ The key conceptual distinction between the 2 focuses on the emphasis on dynamic risk trajectories and phenotyping in the AHA/ACC^2^ framework vs static hemodynamic thresholds in the ESC/ERS approach.^1^ Both guidelines rely on similar clinical, imaging, and biomarker parameters; however, the AHA/ACC^2^ model integrates early signs of deterioration and preshock states, such as measurement of serum lactate and recognition of respiratory indicia, whereas the ESC/ERS algorithm^1^ prioritizes classification based on baseline hemodynamics. The 2026 AHA/ACC guidelines^2^ more explicitly integrate anticoagulation within interventional pathways, including continuation during catheter-directed therapies, whereas ESC/ERS 2019 guidelines^1^ provide less procedural detail. With respect to reperfusion strategies, the AHA/ACC framework^2^ supports consideration of catheter-directed thrombolysis (CDL) or catheter-based embolectomy/mechanical thrombectomy (MT) in selected high-risk or deteriorating patients, whereas ESC/ERS 2019^1^ adopts a more conservative approach, largely reserving these therapies for high-risk patients with contraindications to or failure of systemic fibrinolysis. These recommendations will be likely impacted by an evolving evidence base, including the recent HI-PEITHO randomized trial^3^ and other forthcoming pivotal trials such as PEERLESS II (NCT06055920), PE-TRACT (NCT05591118), and PERSEVERE (NCT06588634). In contrast, ESC/ERS 2019 guidelines adopt a more conservative strategy, mainly reserving percutaneous therapies for high-risk patients with contraindications or failure of systemic fibrinolysis and without distinguishing between CDL and MT, reflecting the more limited evidence available at that time that document was written.^1^

**Figure 1:**
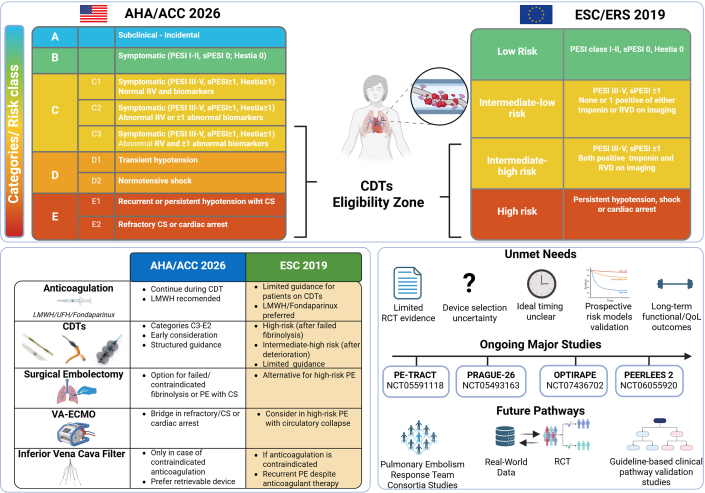
**Comparative Overview of Interventional Strategies and Risk Stratification in Acute Pulmonary Embolism** This figure contrasts the contemporary American Heart Association (AHA)/American College of Cardiology (ACC) 2026 and the European Society of Cardiology (ESC)/European Respiratory Society (ERS) 2019 frameworks for risk stratification and management of acute pulmonary embolism, with a focus on the role of catheter-directed therapies (CDTs) and advanced interventions. The upper panels illustrate differences in risk classification. The AHA/ACC 2026 schema adopts a more granular, phenotype-driven approach, subdividing intermediate-risk PE into categories C1–C3 based on right ventricular dysfunction and biomarker profiles, and further distinguishing hemodynamic compromise (D–E stages). In contrast, the ESC/ERS 2019 model stratifies patients into low, intermediate-low, intermediate-high, and high risk based on PESI/sPESI scores, right ventricular dysfunction, and cardiac biomarkers. The “CDTs eligibility zone” highlights overlapping intermediate-to-high-risk groups where percutaneous interventions may be considered. The lower left panel compares therapeutic recommendations across guidelines. AHA/ACC 2026 provides more structured and earlier consideration of CDTs (particularly in C3–E categories), whereas ESC/ERS 2019 reserves interventional strategies primarily for high-risk PE or clinical deterioration. Differences are also shown for anticoagulation strategies, surgical embolectomy, venoarterial extracorporeal membrane oxygenation (VA-ECMO), and inferior vena cava (IVC) filter use. The lower right panel summarizes current knowledge gaps, including limited randomized controlled trial (RCT) evidence, uncertainty in device selection and timing, and lack of validated prospective risk models and long-term functional outcomes. Ongoing major trials and studies are highlighted, along with future research directions focusing on multidisciplinary PE response teams, real-world data integration, and guideline-based clinical pathway validation. CS = cardiogenic shock; LMWH = low-molecular-weight heparin; PE = pulmonary embolism; PESI = Pulmonary Embolism Severity Index; QoL = quality of life; RV = right ventricle; RVD = right ventricular dysfunction; sPESI = simplified PESI; UFH = unfractionated heparin.

Surgical embolectomy remains an important option for high-risk PE, particularly when thrombolysis is contraindicated or ineffective or in cases of clot-in-transit. The 2026 AHA/ACC guidelines consider it reasonable in category E1 and select D patients, and as an alternative to systemic fibrinolysis to reduce bleeding risk, supported mainly by observational data showing favorable survival and right ventricle recovery even in critically ill patients.^2^ Similarly, the ESC/ERS 2019 document recommends surgical embolectomy primarily in high-risk PE after failed thrombolysis or when contraindications are present.^1^ Both guidelines acknowledge the potential role of venoarterial extracorporeal membrane oxygenation in refractory cardiogenic shock or cardiac arrest, either as a bridge to definitive therapy or as adjunctive support.^1^^,^^2^ However, evidence remains limited and patient selection relies heavily on institutional expertise and multidisciplinary decision-making.^4^^,^^5^

Regarding inferior vena cava filter use, there is strong concordance between guidelines, with both restricting their indication to patients with absolute contraindications to anticoagulation or recurrent PE despite adequate therapy. The 2026 AHA/ACC document further emphasized the preferential use of retrievable filters and the need for structured follow-up to ensure prompt retrieval to minimize long-term complications.^2^ Notably, both guideline documents highlight the uncertain benefit of inferior vena cava filter placement in the context of advanced interventional therapies, reflecting the lack of robust supporting evidence.^1^^,^^2^ The comparison of the 2026 AHA/ACC and ESC/ERS 2019 guidelines highlights important progress in indications for interventional therapies. However, several unmet needs remain. First, high-quality randomized evidence comparing catheter-directed therapies with systemic fibrinolysis or anticoagulation alone is still limited.

Most available data are derived from registries, single arm studies, or small trials and rely heavily on surrogate endpoints, such as right ventricular-to-left ventricular diameter ratio, rather than “hard” clinical outcomes.^6^ For the field to advance, large, adequately powered randomized trials evaluating mortality, clinical deterioration, and patient-centered outcomes are needed. Importantly, the ongoing evolution in PE risk stratification has direct implications for the interpretation of both prior and contemporary clinical trials. Most earlier studies were designed using the ESC/ERS 2019^1^ classification, in which intermediate-risk PE represents a relatively broad and heterogeneous population defined by the presence of right ventricular dysfunction and/or biomarker elevation. As a result, enrolled cohorts often include patients with substantially different hemodynamic profiles and trajectories of clinical deterioration, potentially diluting treatment effects and contributing to low event rates with limited ability to demonstrate mortality benefit. In contrast, the more granular, trajectory- and phenotype-based approach proposed in the 2026 AHA/ACC guidelines^2^ may enable more precise identification of patients at imminent risk of decompensation (eg, Categories D1–D2), who may derive greater benefit from advanced therapies. In such a context, there is also increasing recognition that dynamic risk assessment and clinical trajectory may be more informative than single time point classification, a paradigm that parallels advances in cardiogenic shock, in which staging systems such as the Society for Cardiovascular Angiography and Interventions Shock Classification^7^ and its adaptation to PE-related shock.^2^^,^^8^ Because of this evolving understanding, comparisons across trials should be interpreted with caution, as differences in inclusion criteria may reflect distinct underlying patient phenotypes rather than true differences in therapeutic efficacy. Moving forward, the adoption of standardized, dynamic risk stratification frameworks, alongside prospective validation of the AHA/ACC category-based model, will be essential to improve patient selection, enable phenotype-specific trial design, and enhance the interpretability and comparability of clinical evidence in acute PE.

Second, the optimal timing of reperfusion is not well defined, requiring careful balance between early intervention and stabilization with anticoagulation alone. Furthermore, comparative evidence regarding timing of reperfusion across systemic fibrinolysis, catheter-based intervention, and surgical embolectomy remains limited due to substantial heterogeneity in approaches.^9^ Future studies should clarify whether early intervention in selected patients may improve outcomes compared with a more conservative, anticoagulation-first, rescue-second approach.

Third, procedural standardization in reperfusion is lacking, including thrombolytic dosing for systemic therapy and CDL, and criteria for procedural completion for catheter-based embolectomy/MT, limiting reproducibility and cross-trial comparability. The HI-PEITHO trial^3^ demonstrated a 61% reduction in clinical deterioration without a corresponding mortality benefit, underscoring the challenges of trial design in contemporary PE populations, where overall mortality is relatively low and rescue therapies are widely available and instituted at the first sign of deterioration. In this context, adjunctive tools such as the National Early Warning Score, if prospectively validated, may support serial risk assessment and early identification of deterioration. Additional uncertainties remain regarding the role of adjunctive therapies, including venoarterial extracorporeal membrane oxygenation and pharmacomechanical strategies, as well as the long-term impact of interventions on functional capacity, post-PE syndrome, chronic thromboembolic pulmonary hypertension, and quality of life. Several ongoing trials and studies, including PE-TRACT (NCT05591118), PRAGUE-26 (NCT05493163), and OPTIRAPE (NCT07436702), are expected to address these evidence gaps. Ultimately, progress in the field will depend on integration of randomized and real-world evidence, improved risk phenotyping, and further refinement of adaptive, multidisciplinary treatment pathways.

Overall, despite differences in granularity and timing, ESC/ERS 2019 and 2026 AHA/ACC guidelines certainly appear more convergent than discordant, with differences largely reflecting the time interval and evolving evidence base rather than fundamental philosophical disagreement. Indeed, these guidelines seem to represent a steady progression toward more precision characterization of PE presentation and risk. Forthcoming randomized and nonrandomized contributions to the literature based on this enhanced and evolving understanding of risk are likely to foster greater concordance and precision in routine clinical care and within guideline documents, and as a result, improve outcomes for our patients.

## Funding support and author disclosures

Dr Piazza has received research grants, outside the submitted work, from Bristol Myers Squibb/Pfizer, Janssen, Alexion, Bayer, Amgen, Boston Scientific, and Regeneron, as well as funding support from 10.13039/100000002NIH grant 1R01HL164717-01; and he has also served in advisory roles for Boston Scientific, Amgen, NAMSA, Thrombolex, Penumbra, Bristol Myers Squibb, Janssen, and Nectero. All other authors have reported that they have no relationships relevant to the contents of this paper to disclose.

## References

[1] Konstantinides S.V., Meyer G., Becattini C. (2020). 2019 ESC Guidelines for the diagnosis and management of acute pulmonary embolism developed in collaboration with the European Respiratory Society (ERS). Eur Heart J.

[2] Creager M.A., Barnes G.D., Giri J. (2026). 2026 AHA/ACC/ACCP/ACEP/CHEST/SCAI/SHM/SIR/SVM/SVN Guideline for the evaluation and management of acute pulmonary embolism in adults: a report of the American College of Cardiology/American Heart Association Joint Committee on clinical practice Guidelines. J Am Coll Cardiol.

[3] Rosenfield K., Klok F.A., Piazza G. (2026). Ultrasound-Facilitated, catheter-directed fibrinolysis for acute pulmonary embolism. N Engl J Med.

[4] Mentzel L., Fengler K., Oezkur M. (2025). Mechanical circulatory support in acute pulmonary embolism. Eur Heart J Suppl.

[5] Davies M.G., Hart J.P. (2024). Extracorporal membrane oxygenation in massive pulmonary embolism. Ann Vasc Surg.

[6] Zuin M., Lang I., Chopard R. (2024). Innovation in catheter-directed therapy for intermediate-high-risk and high-risk pulmonary embolism. JACC Cardiovasc Interv.

[7] Naidu S.S., Baran D.A., Jentzer J.C. (2022). SCAI SHOCK stage classification expert consensus update: a review and incorporation of validation studies: this statement was endorsed by the American College of Cardiology (ACC), American College of Emergency Physicians (ACEP), American Heart Association (AHA), European Society of Cardiology (ESC) Association for Acute Cardiovascular Care (ACVC), International Society for Heart and Lung Transplantation (ISHLT), Society of Critical care Medicine (SCCM), and Society of Thoracic Surgeons (STS) in December 2021. J Am Coll Cardiol.

[8] Zuin M., Henkin S., Harder E.M., Piazza G. (2024). Optimal hemodynamic parameters for risk stratification in acute pulmonary embolism patients. J Thromb Thrombolysis.

[9] Zuin M., Piazza G., Barco S. (2023). Time-based reperfusion in haemodynamically unstable pulmonary embolism patients: does early reperfusion therapy improve survival?. Eur Heart J Acute Cardiovasc Care.

